# Practical Observations on the Injurious Effects of Chloroform Inhalation during Labor

**Published:** 1863-09

**Authors:** Robert Johns

**Affiliations:** Chairman of the Midwifery Court, and Examiner in Diseases of Women and Children, Royal College of Surgeons in Ireland, &c.


CHICAGO

MEDICAL JOURNAL.

VOL. VI.] SEPTEMBER, 1863. [No. 9.

### SELECTED.

### PRACTICAL OBSERVATIONS ON THE INJURIOUS EFFECTS OF CHLOROFORM INHALATION DURING LABOR.

By ROBERT JOHNS, A. B., M. D., T. C. D.,
*Chairman of the Midwifery Court, and Examiner in Diseases of Women and Children, Royal College of Surgeons in Ireland, &c.*

As, at the present time, the subject of chloroform inhalation is again *sub judice*, I feel it incumbent upon me to raise my voice against its employment in midwifery, and to lay before my professional brethren my reasons for the adoption of such a course, which I sincerely trust shall have some weight with the unprejudiced, and which may, perchance, call the more serious attention of some, if not of all, of those now too deeply wedded to its use, to the dangerous, and too often fatal, results consequent thereon, in which, if I but even partially succeed, I shall consider myself well repaid.

From experience, repeated observation, and the published, as also the otherwise expressed opinions of those who agree, as well as those who disagree with me upon the subject, I am firmly convinced that chloroform, when inhaled during labor, very fruitfully disposes to hæmorrhage, puerperal inflammation, chest affections, and to other diseases detrimental to health and life, which it aggravates if given during their presence. It also lays the foundation of diseases to arise at a more distant period, and thus increases the mortality in childbed and subsequent thereto. I have known puerperal inflammation frequently to have followed its inhalation, and too often with a fatal result; in fact, some years since, when it was more fashionable, and was given with a more lavish hand, a great mortality obtained amongst the patients of some few men who administered it—so much so that a popular outcry was raised against its employment. In the majority of those cases puerperal fever was the cause of death, which, when thus raised, being, as I firmly believe, always infectious or otherwise communicable, became epidemicised, after which even those who wisely refused the drug, "charmed it never so sweetly," were thus inadvertently, and, in some instances, hopelessly poisoned.

In support of these positions, I shall first refer to the several published Reports of the Dublin Lying-in Hospital. We find, on reference thereto, during the masterships of Drs. Collins and Johnson,* when chloroform was not inhaled, that the mortality was much less than during that of Dr. Shekleton,† when this "pernicious drug was used"—as thus:—In the first report are recorded 16,414 deliveries and 164 deaths, or 1 in 100; in the second, 6,634 deliveries and 65 deaths, or 1 in 102; whereas in the third, 13,748 deliveries are given, and 163 deaths or 1 in 84!! But of these last 13,406 cases were not chloroformed, of which only 133 died, or 1 in 100, and of the remaining 342 who took the drug, 30 died, or 1 in 11!!! If, again, we examine the reported cases of chloroform administration by Simpson and Denham, we shall find that of 245 cases mentioned by the former, 5 died, or 1 in 49; and of 56 by the latter, 5 died, or 1 in 11!! And, by adding all these recorded cases together, we have a mortality on the whole of 1 in 16!!! By again consulting those reports, we perceive that in Dr. Collins' mastership there occurred 97 cases of post partum inflammation, or 1 in 169; in Dr. Johnson's, 62 cases or 1 in 107; but in Dr. Shekleton's, 150 cases, or 1 in 91. Of those 150 cases 20 followed upon chloroform inhalation, or 1 in 17!! and in the remaining 130 cases, in which it was not employed, the average mortality was only 1 in 103. In Denham's report we find 4 cases, or 1 in 14; which, with all the recorded cases, strikes an average of 1 in 16½!!!

We also find that, during Dr. Collins' mastership, puerperal convulsions proved fatal in the proportion of 1 in 6; whereas in that of Dr. Shekleton, when under chloroform, it amounted to 1 in 3!! and in Denham's cases to 2 in 3!!! or, on the whole, to 1 in 2½!!!

*By Drs. Hardy and M'Clintock. †By Drs. Sinclair and Johnston.

It appears that, during Dr. Shekleton's tenure of office, post partum hæmorrhage occurred but once in every 257 cases when chloroform was not used; yet after its inhalation this complication was present in 1 of every 49 cases. In Dr. Denham's report it was present in 1 of every 19 cases; making, on the whole, an average occurrence of 1 in every 39¾ cases.

With respect to the mortality after perforation, the report of Drs. Hardy and M'Clintock shows 1 fatal case in every 6, and that of Drs. Sinclair and Johnston 1 in every 5; but if we go a little below the surface in the latter report, and examine into 99 cases of perforation, all of equal severity and danger, we shall discover that of the 29 cases in which chloroform was inhaled 9 died, or 1 in 3¼; puerperal inflammation occurred 10 times, or 1 in every 3 cases; and hæmorrhage followed in 3 cases, or 1 in every 10; whereas, of the 70 cases in which this drug was not employed, only 6 women died, or 1 in every 12; puerperal inflammation arose only in 3 cases, or 1 in every 23; and in no case did hæmorrhage occur.

Many have testified to the fact that uterine action has been lessened, and even caused to cease, by anæsthetics; as also that their effect on some is not commensurate with the quantity of the drug employed—as thus: a very large amount not having any effect on some, whereas the inhalation of a very small dose, even of a few drops, has produced almost deep coma in others. Dr. Denham says:—"In some, if left to nature, the labor would probably have been completed in a somewhat shorter space of time. The advantages to be gained by chloroform in some cases will not be found an adequate compensation for the loss of power sustained in the muscles of animal or organic life; and, were we to continue its use, I do believe that the patients would remain undelivered for hours, or even days. The cases that apparently require it most—tedious and difficult labors—are those where it often appears to be injurious, by weakening the pains or relaxing the muscles of animal life." Rigby says:—"We meet with cases, every now and then, where chloroform undoubtedly retards labor, and in some cases likely to call for the use of the forceps."

Dr. Robert Lee mentions cases in which "uterine contractions were arrested, requiring the use of the forceps and perforator."

Tyler Smith "has seen chloroform stop labor midway."

In some of the cases recorded by Sinclair and Johnston uterine action was impaired.

My friend, Dr. Young, of Monaghan, says, in a letter to me:

"I believe chloroform in many instances to delay the labor, by causing the pains to come at longer intervals, and rendering the expulsive efforts of the patient less efficient, owing to her insensibility to suffering."

Merriman has mentioned a case in which "the uterus was so paralyzed that it failed to act afterwards."

Snow says:—"It is true that a full dose would, at any time suspend uterine action for a few minutes, or as long as it might be kept up."

Ferguson says:—"Chloroform does not destroy muscular action, because, when under its influence, some expel urine and fæces." Now, from this, his doctrine must be that it increases muscular action; whereas, I take it that it paralyzes the sphincters.

On looking into Drs. Sinclair and Johnston's report, we find "two cases in which version was very difficult; and two others, in which that operation was impossible, where chloroform had been inhaled."

Murphy thus speaks:—"In a case of version, I never experienced so much difficulty in consequence of the strong contractions of the uterine fibres about the child."

Barnes remarks:—"In many cases it does not facilitate the operation of version, the uterus resisting the introduction of the hand."

Puerperal, hysterical and epileptic convulsions—mania, paralysis and insanity have followed on its use. Cases are recorded by Montgomery, Sinclair and Denham, in which puerperal convulsions occurred after its employment. Sinclair gives two cases of hysterical convulsions, in one of which "violent muscular action was induced; restlessness continued for a considerable time after the inhaler was removed."

Murphy states that, in "dentistry, hysterical women have been seized with fits when under its influence."

Snow asserts that "hysterical patients, as soon as they lose their consciousness from the effects of the vapor, are sometimes attacked with a paroxysm of hysteria."

Dr. R. Lee says:—"Epilepsy has been so induced."

Sinclair records one case of epilepsy.

Snow and M. Fix have stated "that persons subject to epilepsy are likely to have a fit brought on by inhaling chloroform."

Ramsbotham "saw three cases of puerperal mania so caused. A friend of his also saw one similar case."

Sutherland "met three other cases similarly produced."

Tyler Smith stated "that he had seen mania from its use."

Parks relates the case of a lady who had chloroform in her third labor. "She, after delivery, complained of violent pain in the head, became delirious, tore the nurse's gown and the bedclothes into pieces, and was perfectly maniacal."

Mr. Banner thus speaks:—"A patient became delirious, and continued so during the day and greater part of the night, after its use."

Haartman "saw a case of headache terminating in paralysis, caused by this drug."

In one of Dubois' published cases, numbness of the fingers, and in another the same condition of the legs, supervened, and had not subsided at the end of twenty-four hours.

In Denham's report I find one case of coma after chloroformic inhalation.

Dr. R. Lee says "that insanity has followed on its employment; that dangerous and fatal peritonitis and phlebitis have been caused by its inhalation."

Two or three of Denham's cases were seized with rigors; and Lee mentions "others with dangerous fits of syncope;" and in this he is borne out by the following, which I find recorded among Denham's cases:—"While inhaling, the pulse became very weak, and she gave no signs of consciousness; and, immediately on the birth of the child, the respiration of the patient ceased, and the pulse became imperceptible; the application of cold water to the face soon revived her, and she went on favorably for several days; but diarrhœa, with extensive inflammation of the mucous membrane of the ileum, set in, and she died on the fourteenth day."

Sinclair and Johnston record nearly a similar case, as thus: "The pulse suddenly became imperceptible, and respiration appeared to have ceased. She subsequently died of phlebitis." And they give another in which collapse occurred, and she died with symptoms of phlebitis.

Tyler Smith says "that he knew two ladies in whom a few drops of chloroform, at any time, would produce repeated fainting."

I am acquainted with a lady who, some time since, had a very severe attack of syncope from taking only five drops of chloroform in a draught.

Dr. Barnes stated—"That he had himself given chloroform to facilitate the extraction of an adherent placenta, and had witnessed such exceeding prostration for eight hours afterwards, as to make him, and another practitioner who assisted him, apprehensive of the instant death of the patient."

Many are of opinion that the inhalation of chloroform predisposes to laceration of the perinæum; indeed, some of the published cases would tend to favor this idea. In Sinclair and Johnston's report, we find that, in the recorded cases, it occurred once in 27 cases; and when not employed, the accident happened only once in 93 cases. In the same work we find 3 cases of chest affection aggravated by this means, two of which succumbed. Dr. Ringland, one of the Masters of the Coombe Eying-in Hospital, in reply to a letter from me, thus writes:—

"I have seen chloroform frequently used in puerperal convulsions, and have used it myself in connection with the practice of the Coombe Lying-in Hospital; and the conclusion I have come to is, that I will never again use it, or sanction its use, in puerperal convulsions. I have observed that, however satisfactory its employment may appear at the time, it has been almost invariably followed by bronchitis, within 48 hours, and that the patients have sunk rapidly under the latter affection. I have seen this so frequently that I cannot but look on chloroform and bronchitis, under the circumstances I have named, as cause and effect; and the mortality from the subsequent bronchitis, as the actual result of the employment of chloroform.

Ramsbotham relates the case of "a lady who was seized with dyspnœa, with excessive lividity of the face, and all the signs of engorgement of the lungs and heart, and died in convulsions six hours after."

Murphy has published a case nearly similar; he also admits "that vomiting, nausea and headache sometimes follow on its use." Nausea and vomiting were also present in some of Denham's cases.

Rigby states, "that intense headache, and even vomiting are consequences of its use."

I occasionally use a blistering fluid which contains chloroform, and if I am not very cautious during the *minute* I am employing it, I am certain to suffer from sick headache for the remainder of the day. Not long since, severe vomiting followed upon the inhalation of chloroform, during the operation for vesico-vaginal fistula, in one of our city hospitals; and, in spite of all remedies, lasted for six days. It is needless to say that the operation, in consequence thereof, failed. I have so often seen this effect of the drug that I always object to its use in operations requiring the employment of sutures upon the female genitals. Thus, it is evident that such a complication existing after labor would, like severe cough, predispose our patient to inflammation in parts, for whose restoration to health absolute rest is required.

Parks gives the case of a "lady in whom, after chloroform inhalation, flooding came on to a fearful extent, and incessant sickness. He managed to extract the placenta; and, owing to the feeble contractions of the uterus (and this latter condition, he is confident, it often produces), he was kept grasping it for four or five hours; the vomiting continued for eight hours without intermission; the headache remained for weeks."

Tyler Smith "believed that post partum hæmorrhage and retention of the placenta occurred more frequently after its use than without it."

Montgomery was of opinion "that it predisposes to retained placenta and hæmorrhage."

My friend, Dr. Young, before alluded to, says:—"I have blamed it for causing a longer detention of the placenta, and for occasional after-hæmorrhage, owing to the lazy and inefficient contraction of the uterus. After its use opiates have very little effect; even very decided doses, in any form, have not been followed by that tranquility I had hoped for, in that violent pain which I have so often found to follow operation when chloroform had been used."

Murphy speaks of making pressure on the uterus to expel the placenta, in two cases, after chloroform.

Denham had one case of retained placenta after its employment. He says:—"We had no reason to think that chloroform predisposed to hæmorrhage; on the contrary, we were impressed with the idea that the number of hæmorrhagic cases where it had been given were rather below than above the average in ordinary practice." This statement does not accord with my experience, and I should be sorry to think that hæmorrhage so frequently complicated labor, "in ordinary practice," as once every *nineteen cases*, as shown by his report. Some of the loudest advocates for chloroform inhalation in labor have, in order to counteract its deleterious effects upon uterine action, recommended the co-administration of ergot of rye; which practice reminds me of the astute physician who, to be sure to hit his patient's disease, prescribed for him the combination of a stimulant with a sedative, and a purgative with a tonic. But I hold that there is a more serious objection than this to the wholesale use of ergot; for we cannot conceal from ourselves the fact that its administration, even in appropriate cases, is not always innocuous. Some years since the following case came under my knowledge:—Ergot was given to an unmarried lady to facilitate the birth of her first child, before her father, who was ignorant of her condition, had returned home to his dinner. The child was rapidly expelled, but sloughing, to a frightful amount, followed, and placed her life in jeopardy for days. And who has not seen the child sacrificed by it? For this reason, it has now-a-days become almost an axiom not to leave a female undelivered for a longer period than two hours after its employment. I believe that ergot of rye, in some cases, causes incarceration of the placenta and hæmorrhage, and in others, sinks the patient; the uterus, after its use, often remains large and uncontracted for days, which state not unfrequently terminates in imperfect involution of the uterus and its consequences; which last effect chloroform also produces. Many believe that ergot, besides destroying the child at the time of its birth, acts sometimes otherwise deleteriously upon it, by inducing disease—to do so at a shorter or longer subsequent period—or to reduce it to a state to which death would be preferable.

Dr. Catlet, in the 57th volume of the *Edinburgh Medical Journal*, page 83, states that ergot of rye, when given during labor, causes puerperal convulsions, hour-glass contraction of the uterus, and infantile hydrocephalus. Amongst the cases of the last, I find one in which "symptoms of meningeal inflammation were developed on the 19th day, and the child died in convulsions, with coma, on the second day following." And in another, "the symptoms of cerebral derangement set in suddenly on the 21st day, and the child died on the third day of the attack, in convulsions."

Dr. Beatty, in a paper "On the Influence of Ergot of Rye on the Fœtus in Utero," published in the 25th volume of the *Dublin Medical Journal*, page 201, amongst other cases after its use in labor, gives the following:—"Case 7. The child had convulsions for three days after its birth." "Case 9. The child had convulsions for 48 hours after birth. They then subsided but left the child in a state resembling paralysis, with occasionally a convulsive motion of the muscles of the face and limbs, and fixed strabismus. No treatment seemed to have any effect upon this condition. Twenty days after its birth the following report was taken:—'This child has remained in a state of insensibility up to the present time; the strabismus has lately disappeared, but it seldom opens its eyes. The limbs are apparently powerless. It makes no effort to suck, but it swallows breast-milk with difficulty when put into its mouth. The difficulty is increasing; the bowels act naturally.' In this state the child lingered on until the 25th day, when it died." Case 12. This child he first saw when three years old; "it then had an idiotic countenance, and was never free from spasms and palsy, commencing from its birth."

Cusack and others have also testified to the deleterious effects of this drug upon the cerebro-spinal system of the infant.

Dr. Snow says that "chloroform is a volatile spirit, and that half an hour after its application no traces of it could be found in the system."

Now, in refutation of this assertion, Dr. Ramsbotham mentions the case of "a lady who, for four or five days after its use, could not get rid of the smell."

Dr. Aveling speaks of "a lady who had chloroform in three labors, all of whose children, when unwell, had for years afterwards the smell distinctly off their breaths. This lady would never take it again."

In a monograph by me, on "Blistering the Os and Cervix Uteri," published in the May number of the *Dublin Quarterly Journal*, of the year 1857, cases are mentioned of females having had the smell of chloroform off their breaths, evident to their friends as well as to themselves, and of others having experienced its taste, lasting in both instances, for days after the blistering fluid containing that drug had been employed.

When sulphuric ether was first employed as an anæsthetic in this country, a medical student inhaled it as an experiment in this city, and the smell of it was evident off his breath, to any one who spoke with him, for nearly a week after its employment.

Dr. Jackson (an American) thus writes upon the subject:—"When chloroform is inhaled into the lungs, the oxygen is abstracted from the blood, and, combining with the formyle, makes formic acid, while the chlorine combines with the blood as a substitute for oxygen. Thus a portion of the blood becomes chemically changed, disorganized, and rendered unfit for its vital functions. I have now a phial of blood, taken from a young lady killed by the inhalation of pure chloroform, before me, it having been kept in my office, exposed to temperatures from the freezing point to above 80°, for more than six years, and yet it has not decomposed, nor has a single blood-globule settled to the bottom of the phial, nor has the color changed in the least." It has been denied that females, when under the influence of chloroform, make use of improper and indecent language. Now, I never shall forget the case of a lady I saw, in consultation, a couple of years ago, with an hospital surgeon, who, when chloroformed, threw her arms around him in the most endearing manner, and made use of language which would make her blush if in her senses, of which, I hope sincerely, she was never made cognizant.

Denham says :—" There are cases in which chloroform appeared to be not only useless, but, when persevered in, positively injurious." And again :—" In giving chloroform we incur a certain amount of present danger, and perchance of remote ill effects."

Dr. Robert Lee, in reply to a letter from me, says :—" I could give you a great number of cases in which chloroform was not only injurious, but fatal."

Dr. Gream said :—" He agreed with Dr. Lee in saying that we were quite unacquainted with *one-tenth* of the evil effects which had resulted from the use of chloroform, particularly in Scotland."

Dr. Duncan, in a letter to Dr. Lee, thus writes :—" Your case of chloroform death in midwifery, is, to the best of my belief, not the only one in Scotland. I was called, too late, to a case which died suddenly while taking it in *small quantity*."

Dr. Campbell, of Ayrshire, records another case of death in labor from its use. Mr. Carter says " that in two cases its effects would appear to have been pernicious."

Prof. Faye, of Christiana, has also recorded a fatal case of labor after its use.

Dr. Barnes says :—" In ordinary forceps cases chloroform certainly is not required, either to facilitate the operation or to allay pain." Indeed by its use in such cases we lose one very valuable indication by our patient's want of sensibility. Dr. Chas. Kidd evidently does not consider its use devoid of danger, as he advises the physician who administers it " *always* to carry in his pocket a portable galvanic chain or battery." Drs. Kidd and Richardson are reported as having seen many deaths after its employment; and the former gentleman " to have seen about 300 cases restored to life or rescued after they had been pronounced dead." I would ask, in the name of common sense, is it within the bounds of reason to believe that a medicine can be employed innocuously with the pregnant female, when confessedly its use has often been followed, not only by dangerous, but even fatal results under other circumstances, as testified to by Drs. Kidd and Richardson, amongst many others, as also by almost every periodical we take up? Dr. Show, in speaking of his imagined advantage of chloroform over opium in version cases, thus writes :—" If 50 or 60

drops of laudanum were given, the patient remained under its influence, more or less, for 48 hours." Now, in this I must join issue with the Doctor, for I am, and have been for years, in the habit of giving such, and even much larger doses in those cases, as also in hæmorrhage, and I never yet saw such a result, or one at all approaching to it. We have been told that across the Tweed death has not, in any instance, followed upon the inhalation of chloroform in labor, whereas some have been since recorded; and not very long ago I was informed, by more than one physician practicing in Scotland, that many have so occurred there, but not made public, yet well known to the profession. It is also a fact that some who have written favorably on its use have since changed their opinions, but have not said so publicly; and some give it only in name, or as has been styled *a la Reine.* The following is so apposite here that I cannot avoid quoting it from Denham:—"That chloroform may be, and sometimes is, given for the purpose of amusing patients, and making them believe that they are saved from a vast amount of pain, when in reality they have scarcely inhaled a single breath of it, I doubt not."

We very frequently see safer and better recoveries after tedious and painful than after rapid and painless labors, and the latter are not the less likely to be seriously complicated; indeed, in former days, when, happy for the parturient female, chloroform was unknown, and when meddlesome midwifery was strongly reprobated, such an opinion was entertained. *Apropos*, I have two patients—one the mother of five, the other of four children—who always have rapid and, I may say, painless labors, but which are invariably followed by alarming hæmorrhage, by no means an unusual occurrence, as already shown, after chloroform inhalation, besides being admittedly a fruitful predisposing cause of puerperal inflammation. In the employment of anæsthetic agents during instrumental delivery we deprive ourselves of a very valuable indication in the loss of our patient's sense of feeling, which the following cases forcibly illustrate; for had such means been resorted to in either, it must be evident to all, even to the most sceptical, that the consequences should have been most disastrous:—Mrs. D. had a very tedious labor with her first child. When about 36 hours in labor, the os uteri was found thinned and spread tightly over the head of the child, dilated to about the size of a shilling, but directed obliquely backwards and upwards, so located as only to be found by the well-educated and practiced finger. Her medical attendant, having failed to discover the real state of matters, took it for granted that he only felt the head, which had passed through the fully dilated os, and proceeded, without further delay, to deliver her with the forceps; but from the great pain which she experienced from the application of its blades on the head so clothed, he was obliged to desist; and, being much alarmed, he sought for further assistance, after which the nature of the case was discovered, when of course, all interference was given over for the time, but eventually destructive instruments were had recourse to. The other was the case of Mrs. M., very similar to the former; but the perforator was the instrument employed, which the medical gentleman pushed into the cervix expanded over the head, when her piercing cries and some slight bleeding caused him to look more narrowly into the state of the parts. She was, however, afterwards naturally delivered, and had a good recovery.

At page 333 of the *Dublin Quarterly Journal of Medical Science*, for May, 1849, in the late Dr. Montgomery's essay upon "The Indiscriminate Administration of Anæsthetic Agents in Midwifery," we find a somewhat similar case recorded, in which the medical man mistook the attenuated anterior section of the cervix uteri for the membranes, which he was endeavoring to perforate with his nail, when the lady's cries arrested him.

Even though it were possible to divest chloroform of its dangers, it does not, as has been already shown, always produce the advantages expected from its use, as in version; for indeed not a few instances have been recorded of its having been an impediment to this operation, which in some cases could not be overcome. I cannot see any advantage derivable from the inhalation of this poisonous drug in cases of retained placenta, as generally such a complication is caused by inaction of the uterus; and our object, therefore, ought to be to induce uterine action, surely not further to paralyze it. Such treatment reminds me of a case which I was called to see 20 years ago. The placenta had been retained for 6 hours, and some draining was going on. The lady's medical adviser was looking on very complacently, and dosing her with tartar emetic. Of course there was not any difficulty in the extraction; but puerperal inflammation set in on the second day, from which she eventually but slowly recovered. Every practical man hails after-pains as salutary, especially after quick and painless labors, and would not dream of interfering with their wholesome action, unless very severe, for some hours after delivery ; yet those misguided chloroformists think nothing of interfering with that safe action at times when the advent of hæmorrhage would complicate matters more seriously. The other objections to its use at other times, under certain circumstances, are equally admissible here. I think I have now demonstrated that chloroform inhalation is far from being a safe remedy in childbed, and should not then be employed.—*Dublin Quarterly Journal of Medical Science*, May, 1863.

